# Sleep and Sleep Disorders in Rare Hereditary Diseases: A Reminder for the Pediatrician, Pediatric and Adult Neurologist, General Practitioner, and Sleep Specialist

**DOI:** 10.3389/fneur.2014.00133

**Published:** 2014-07-17

**Authors:** Natan Gadoth, Arie Oksenberg

**Affiliations:** ^1^Sleep Disorders Unit, Loewenstein Rehabilitation Center, Raanana, Israel; ^2^Department of Neurology, Mayanei Hayeshua Medical Center, Bnei Barak, Israel; ^3^Sackler Faculty of Medicine, Tel-Aviv University, Tel-Aviv, Israel

**Keywords:** sleep apnea, breathing, genetic, metabolic, neuromuscular, upper airway

## Abstract

Although sleep abnormalities in general and sleep-related breathing disorders (SBD) in particular are quite common in healthy children; their presence is notably under-recognized. Impaired sleep is a frequent problem in subjects with inborn errors of metabolism as well as in a variety of genetic disorders; however, they are commonly either missed or underestimated. Moreover, the complex clinical presentation and the frequently life-threatening symptoms are so overwhelming that sleep and its quality may be easily dismissed. Even centers, which specialize in rare genetic-metabolic disorders, are expected to see only few patients with a particular syndrome, a fact that significantly contributes to the under-diagnosis and treatment of impaired sleep in this particular population. Many of those patients suffer from reduced life quality associated with a variable degree of cognitive impairment, which may be worsened by poor sleep and abnormal ventilation during sleep, abnormalities which can be alleviated by proper treatment. Even when such problems are detected, there is a paucity of publications on sleep and breathing characteristics of such patients that the treating physician can refer to. In the present paper, we provide an overview of sleep and breathing characteristics in a number of rare genetic–metabolic disorders with the hope that it will serve as a reminder for the medical professional to look for possible impaired sleep and SBD in their patients and when present to apply the appropriate evaluation and treatment options.

## Introduction

Sleep disorders cause significant morbidity in the general population. While an impressive progress has been made in the recognition and treatment of such disorders in adults, the same disorders which affect as many as 30% of healthy children are still under-recognized (1).

Inborn errors of metabolism (IEM) and non-metabolic genetic disorders manifest mostly during early childhood with progressive neuromuscular, skeletal, and/or neuro-cognitive abnormalities. The affected children suffer frequently from abnormal sleep accompanied by disordered breathing. It is conceivable that under-recognition of sleep problems in this particular young population is much more pronounced due to the rarity and complex symptomatology of such disorders.

Sleep and breathing are tightly linked. Human sleep is induced and modulated by specialized central neuronal networks; however, recent evidence suggests that there are also local areas of the brain involved in sleep–wake modulation (2). Normal breathing is regulated by central and peripheral chemo- and mechano-receptors that are responsible for the reduction in the slope of the ventilatory responses to hypoxia and hypercapnia during sleep as compared to wakefulness. As a result, the minute ventilation drops progressively reaching its minimum during the phasic stage of rapid eye movement (REM) sleep (3).

Sleep-related breathing disorders (SBD) are quite common in the general population. Snoring and obstructive sleep apnea (OSA) are the main SBD for which children and adults are now-a-days referred to sleep disorders centers and as such are considered the “bread and butter” of sleep medicine. Nevertheless, children and young adults with hereditary disorders associated with SBD are only rarely evaluated by such centers. Thus, even in a sleep center with special interest in hereditary disorders, very few such patients are expected to be evaluated. The specialist who is dealing with a child or a young adult afflicted with one of those disorders is so busy taking care of his young patient’s serious and complex medical and behavioral problems that the issue of sleep quality is either overseen or masked by the severity of other symptoms. This is also true in the case of the general practitioner and the adult neurologist when they “takes over” the care of such a child as he gets older but has not “grown out” of his sleep problems.

Out of the numerous metabolic/genetic disorders, we have elected to review in-brief those with well-documented impaired sleep as well as SBD in an attempt to provide the primary physicians and the specialists taking care of such patients as well as the sleep practitioners with a relatively “quick reference” to the nature of the sleep disorder that their patient may suffer from. We hope that in this way, we will help the interested reader to apply the appropriate method of evaluation and provide treatment, which may improve the life quality, if not prolong the shortened life expectancy of their patients.

## Inborn Errors of Metabolism

Inborn errors of metabolism are rare genetically determined biochemical disorders caused by a single gene defect that result in a deficient enzyme or protein and consequently lead to characteristic clinical and biochemical abnormalities. The diseases caused by IEM vary considerably in their clinical and pathological aspects. They commonly present during infancy, early childhood, and more rarely during early adulthood. The clinical diagnosis of IEM may be easily reached by physicians familiar with those disorders, although a biochemical confirmation is required to establish a sound diagnosis, which in a number of those disorders may lead to successful therapy. In addition to the well-known clinical and laboratory manifestations of those disorders, there are also associated and frequent sleep abnormalities, which have not gained sufficient interest and insight. Many individuals with IEM are retarded, restless, and difficult to communicate. Those features may lead quite often to the conclusion that the patients are “problematic sleepers” and there is no need for costly and in many patients an almost impossible task to study their sleep by nocturnal polysomnography (NPSG) at the sleep center or at home using a portable sleep recording device. Moreover, there is a tendency to regard retarded people, many of them with IEM, as impaired sleepers without the need to look for the precise cause and nature of the disorder. Considering the fact that the majority of the disorders, which we have included in this review are associated with skeletal anomalies affecting the chest wall and the upper airway (UA) tract, it is not surprising that SBD is the most common form of sleep disorder in those patients.

## Storage Diseases

### Mucopolysaccharidosis

The mucopolysaccharidoses (MPSs) consist of rare heterogeneous genetically determined lysosomal storage disorders caused by deficient or inactive enzymes necessary for the breakdown of glycosaminoglycans (GAGs). GAGs are complex carbohydrates composed of repeats of disaccharide units, which form long unranked polysaccharides. As a result, GAGs are stored within the lysosomes and cause systemic organ damage. Seven different types of MPS, i.e., I, II, III, IV, VI, VII, IX and additional subtypes of III and IV are known. All types are autosomal recessive (AR) except MPS II, which is autosomal dominant (AD) (Hunter syndrome) (4). The world wide prevalence of MPS is 0.6–5:100,000 and the life expectancy is about 20 years (5). The cardinal abnormalities are musculoskeletal and cardiovascular in the form of systemic and pulmonary hypertension and congestive heart failure. Another clinical feature is moderate–severe cognitive impairment, mainly in MPS III and to a lesser extent in MPS I, II, and VII, which is related mainly to accumulation of GAGs in the perivascular spaces of the brain. There is also neuropathological evidence of ongoing neurodegeneration in several subtypes of MPS (6). UA obstruction is common in all forms of MPS due to adenotonsillar enlargement, large and protruded tongue, reduced retropalatal and retroglossal space, narrow trachea and nasal airway, short neck and small thoracic cage. Those anatomical changes are caused by excessive accumulation of GAGs in the UA. Recently, analysis of the UA in children with MPS with the acoustic reflection method showed significant UA obstruction due to reduction of the minimal cross-sectional area of the subglottic region, the narrowest part of the UA. This finding was associated with an increase in airway resistance (7). Those anatomical features are responsible for the high frequency of OSA, which deleteriously affects cardiovascular morbidity in MPS. Unfortunately, complete PSG studies were reported in a small number of patients. Nashed et al. (8) have studied 14 children and adolescents, most of them with MPS I, with NPSG trying to document not only the basic characteristics of nocturnal sleep and breathing features but also to evaluate the influence of enzyme replacement therapy (ERT) given to three of those patients. OSA was found in 7 (64%) of the 11 untreated children (mean age 5.2 years) and was scored as moderate–severe in 6 despite prior adenotonsillectomy. In the three children who receive ERT, a significant reduction in apnea hypopnea index (AHI) was noticed. Central sleep apnea was present in only one patient with MPS I. In a quite recent home sleep study on 19 patients with various subtypes of MPS, 18 were found to suffer from OSA (11 with severe OSA) (9). Thus, early recognition of OSA and proper treatment in those patients may reduce the high cardiovascular mortally and perhaps improve their daily vigilance.

### Glycogen storage disease

This group of disorders is caused by functionally defective enzymes involved in either breakdown or synthesis of glycogen. Glycogen storage disease (GSD) affects mainly the liver and muscle. Out of the presently recognized 11 subtypes of GSD, 8 affect muscles and are entitled “muscle glycogenoses.” Of those, GSD II, known also as Pompe’s disease (PD), (OMIM #232300), affects significantly respiratory muscles and is associated with SBD. PD is an AR disorder caused by a deficiency of the lysosomal enzyme α-glucosidase (acid maltase) resulting in lysosomal accumulation of glycogen in almost all tissues, however, the first clinical manifestations are frequently due to dysfunction of cardiac and skeletal muscles. The three phenotypes of the disease are determined by the amount of residual acid maltase activity, which is dismal in the fatal infantile form. In the adult onset form, the residual enzyme activity is higher; therefore, the resulting disorder is relatively benign. There is also an intermediate childhood onset form. Respiratory muscles are involved in all phenotypes. In the infantile form, pronounced obstructive hypertrophic cardiomyopathy and skeletal muscle weakness and atrophy are caused by storage of glycogen within muscles and spinal motor neurons. The disease course is rapidly progressive leading to fatal cardio-respiratory failure before the children reach their first birthday. The intermediate childhood form presents with delayed motor milestones detected at the age of 6–12 months when progressive proximal muscle weakness without signs of cardiomyopathy are noted. The main cause of death is respiratory failure (10). In the relatively mild adult form, proximal muscle weakness may not be evident until the age of 20–60 years while respiratory problems appear earlier and are much more prominent. It is typical for those patients to be able to carry their heavy oxygen tank on their way to the initial neuromuscular evaluation. Respiratory failure is progressive in all forms of PD. In a recent retrospective study, 17 patients aged 0.5–17.5 months who suffered from the infantile form, underwent at least a single NPSG study. Six had mild and one had moderate OSA. The mean sleep efficiency was 73%. Interestingly, even in infants without symptoms of SBD, hypoventilation and OSA were recorded. This finding led the authors to recommend routine PSG in every infant with PD (11). Only 30% of adult patients with PD complain initially of daytime somnolence and morning headaches (12), and the main PSG findings are related to severity of daytime somnolence. In four patients with childhood onset PD, Nabatame et al. (13) have recorded hypopneas and apneas during NREM sleep with accentuation during REM sleep regardless of the presence of muscular symptoms. Three patients with NPSG proven SBD received non-invasive intermittent positive pressure ventilation during sleep over 2 years. Although SBD resolved and life quality improved, the impaired respiratory functions were not similarly affected. Patients with the adult form had abnormal AHI, which markedly decreased after institution of mechanical ventilation. Only a weak correlation was found between respiratory and muscle functions, which was relatively preserved while respiratory functions were failing (14). Worsening of SBD during REM sleep is not a specific feature of PD being common in variety of neuromuscular disorders (15).

### Aspartylglucosaminuria

Aspartylglucosaminuria (AGU) (OMIM #258406) is a rare and severe AR lysosomal storage disorder that affects the central nervous system, skeleton, and connective tissue. The main characteristic clinical feature is mental deterioration. AGU is caused by deficient activity of the lysosomal enzyme glycosylasparaginase due to a mutated aspartylglucosaminidase gene located on chromosome 4. As a result, series of glycoasparagines with an aspartylglucosamine moiety at the reducing end are accumulated in tissues and body fluids (16). Premature death occurs at the age of 35–40 years (17). Adolescent patients suffer from bouts of inability to fall asleep accompanied by confusion lasting few days (18). About 25% of the patients suffer from maladaptive sleep behavior, which was attributed to mental retardation (19). We could not find accounts of PSG studies in the English written literature. A single sleep questionnaires study of 81 patients disclosed difficulties in sleep setting in the young patients and severely fragmented night sleep in older patients (20). Although the reason for the disordered sleep pattern is not clear, the presence of hypointense appearance of the thalami on T2-weighted MRI images (21), which may be caused by the metabolic impairment, can perhaps explain this particular sleep pattern.

### Neuronal ceroid lipofuscinosis

This is a group of heterogeneous inherited progressive degenerative diseases, which affect the brain and retina due to lysosomal accumulation of ceroid-lipofuscin, an extremely inert and stable lipopigment that displays typical ultrastructural patterns. Mutations in nine different genes are known to cause neuronal ceroid lipofuscinosis (NCL). The age of clinical onset varies from birth to adulthood. The etiology of NCL remains elusive.

The juvenile onset form (CLN3), known also as Batten–Mayou syndrome (OMIM #204200), is one of the most common childhood onset neurodegenerative disorders in all ethnic groups except in Jews (22). The main clinical features are visual loss first noticed at 7 years of age, which progress to total blindness at the age of 13–14 years, cognitive decline, and seizures. Life span is significantly shortened and disordered sleep is quite common. According to sleep questionnaires, 55% of 42 patients were found to suffer from difficult sleep stetting, numerous awakenings, and nightmares (23). In a clinical and NPSG study of 28 patients, 6–27 years of age, total sleep time, sleep efficiency, and REM sleep duration were decreased while the percentage of sleep stage 1 and the number of awakenings were increased (24). The fact that all patients were practically blind raised the possibility that the findings were secondary to poor vision. This assumption was based on the fact that sleep disturbances and abnormal melatonin rhythm are more common in blind children without light perception (LP) than in those with LP (25, 26). Although melatonin rhythm was not studied in the NCL cohort mentioned above, the sleep structure abnormalities documented were irrespective of the degree of severity of visual impairment.

### Wilson’s disease

This AR disorder of copper metabolism (OMIM #277900) is manifested by systemic storage of copper due to deficiency of a membrane-bound copper transporting P-type ATPase. Patients with Wilson’s disease (WD) carry hundreds of mutations in the ATPB7 gene located on chromosome 13 (27, 28). As a result there is a decreased incorporation of copper into ceruloplasmin, the copper binding protein, leading to free copper accumulation especially in the liver, cornea, and nuclei of the putamen and globus pallidus. Neurological symptoms are present in about 40% of the patients and consist of a variety of progressive involuntary movements that appear during childhood and very rarely after the age of 40 years. Disordered sleep is quite common. Neshimalova et al. (29) used sleep questionnaires in 55 adults with confirmed WD and performed NPSG followed by Multiple Sleep Latency Test (MSLT) in 24. The results were compared to those obtained from 25 healthy age- and sex-matched controls. Forty-one (80%) of the patients reported daytime tiredness, excessive daytime sleepiness, cataplexy-like episodes, and poor nocturnal sleep. In the majority of the patients, those problems appeared concomitantly with the clinical onset while only three patients stated that their sleep problems started before they became symptomatic. Interestingly, the subjective sleep complaints were unrelated to hepatic or neurological morbidity. On NPSG, sleep efficiency, total sleep time, and latencies to sleep stage 1, 2 and NREM sleep were decreased. Periodic limb movements (PLM) were recorded in 40%. On MSLT, shortened or borderline sleep latencies were found in a third of the patients. Sleep onset REM periods on NPSG or MSLT were not found. Once more, the results of nocturnal NPSG and MSLT could not be related to disease severity. In a study from India (30), the clinical and NPSG characteristics of sleep were studied in 25 patients. Total sleep time, sleep efficiency, duration of slow wave sleep, and REM sleep percentage were reduced while sleep latency was prolonged. The patients who were on de-coppering treatment had prolonged REM latency and mixed apnea. The differences between the two-mentioned studies may imply the presence of genetic heterogeneity, different treatment regimens, and/or differences in the extent of copper storage and its location in the central neural pathways involved in normal sleep.

### Friedreich ataxia

Friedreich ataxia (FRDA) (OMIM #229300) is one of the commonest AR ataxias affecting 1:50,000 births (31). It is characterized by progressive loss of sensory neurons in the spinal cord and dorsal root ganglia associated with ataxia, sensory neuropathy, kyphoscoliosis, cardiomyopathy, and premature death. FRDA is caused by expansion of TAA triple nucleotide repeats within the intron of the FRDA gene (Frataxin) located on chromosome 9. Fatigue is a frequent complains in FRDA (32). A recent NPSG study of 21 patients with an Epworth Sleepiness Scale of >8 showed that 21% of the participants suffered from OSA. The authors suggest that OSA in FRDA is related to disease duration and the presence of reduced respiratory muscle strength in conjunction with scoliosis and poor posture (33).

## Chromosomal Anomalies

### Rett syndrome

Rett syndrome (RTS) (OMIM #312750) affects 1:10,000 female infants, 6–18 months of age and manifests with loss of acquired speech, stereotype “hand clapping,” deceleration of head and brain growth, autistic behavior, emotional withdrawal, lack of eye contact, seizures, and severe “dysautonomia” in the form of respiratory, cardiac, and gastrointestinal dysfunction. About 25% of the affected girls die prematurely of cardio-respiratory failure (34). More than 95% of the girls show a loss-of-function mutation in the gene encoding Methyl-CpG-binding protein 2 (MeCP2) located on chromosome X. Over 200 different mutations are known to be present in patients with RTS. Breathing abnormalities are common and severe enough to be included in the clinical diagnostic criteria of the syndrome. The characteristic abnormalities are breath holding spells and apneas coupled with subtle bradycardia followed by exaggerated tachycardia. Those are pronounced when awake and to a much lesser extent during sleep (35). In 30 affected girls, PSG showed only subtle breathing abnormalities during REM sleep in contrast to marked breathing abnormalities when awake. This unexpected finding suggests that in RTS, an imbalance may exist between the behavioral and metabolic control of respiration. During wakefulness, marked breathing abnormalities may be caused by impaired cortical respiratory control (behavioral). Indeed, during wakefulness, the girls have bouts of hyperventilation followed by apnea (post-hyperventilation apnea, PHA), which occur after a happy and/or exciting event. During the event, they appear comfortable in spite of being cyanotic. This peculiar behavioral pattern is similar to that observed in children with congenital central hypoventilation syndrome (CCHS) whose apneic episodes were coined “happy hypoxia” (36). Considering the marked obsessive characteristics of girls with RTS, it seems logical that the attacks of PHA are also obsessive (“voluntary”). This may explain the contrast between daily severe breathing abnormalities when awake and the almost normal breathing when asleep. Although PHA is only rarely seen in healthy individuals, it is often present when the cerebral cortex control is subdued by sedation, anesthesia, age-related frontal lobe dysfunction and dementia. Interestingly, this unique breathing pattern is so common in RTS while it is very rare in other forms of mental retardation and other types of autistic disorders. A clear-cut explanation for its presence in RTS is still in need.

### Down syndrome

Down syndrome (DS) (OMIM #190685), the most frequent form of mental retardation, is caused by trisomy of all or a critical portion of chromosome 21. A large number of the characteristic features of DS such as midfacial hypoplasia, mandibular hypoplasia, glossoptosis, abnormally small UA, superficially positioned tonsils; relative tonsillar and adenoidal encroachment and hypotonia of the UA are all known risk factors for OSA. Thus, it is not surprising that OSA may be present during early infancy and contribute to the developmental delay of the affected infants. Indeed, among a cohort of 108 patients 1–18 years of age, 64.7% had SBD (37). OSA is present in 30–55% of children with DS and reaches a peak of 94% during adulthood (38). The worsening of OSA is related not only to age and obesity but also to increased rate of DS associated hypothyroidism. Patients with DS compared to normal developing age-matched subjects, have greater bedtime resistance, sleep anxiety, increased short awakenings from night sleep, bruxism, night talking, and daytime sleepiness. Considering the high frequency of tonsillar and adenoidal hypertrophy in children with DS, it is somewhat surprising that tonsillectomy and adenoidectomy improved some parameters of OSA, but the outcome was inferior to that of non-DS children. This fact implies that besides the enlarged tonsils and adenoids, the additional bony and soft tissue anomalies of the UA tract mentioned above may play an important role in the high frequency of SBD in DS. This assumption is supported by an earlier NPSG study on 10 institutionalized patients aged 6–32.2 years with a mean body mass index (BMI) of 29.4 in whom the authors excluded UA pathology. The outstanding finding was the marked preponderance of central apnea in this particular group of patients (89.4% central and 9.4% obstructive) (39). The discrepancy between the results of the earlier and recent study mentioned above may reflect the selectivity bias as well as the small sample size of the earlier study.

### Fragile X syndrome

Fragile X syndrome (FXS) (OMIM #300624) is a X-linked syndrome considered the most common inherited cognitive–behavioral disorder in boys while girls are very rarely affected. The silenced mutated fragile X mental retardation-1 (FMR1) gene product is a cytosine–guanine–guanine (CGG) triplet expansion with ≥200 repeats. Animal studies have indicated that normal sleep–wake cycle is significantly blunted in the absence of fragile X mental retardation (FMR) protein (40). Sleep problems are quite frequent in the affected children and may be noticed in many before the age of 3 years. Those consist of difficult night setting, restless interrupted sleep, early awakening, and excessive daytime sleepiness (41). Unfortunately, only few subjects with FXS underwent NPSG, because of the difficulties of performing such a study in retarded children with difficulties in maintaining sufficient nocturnal sleep. In a cohort of 14 boys with a mean age of 13.1 years, reduced time in bed, higher percentage of stage 1, NREM, and a lower percentage of REM sleep were documented.

Interestingly, when compared to children with DS who are also mentally retarded but have a quite docile behavior pattern, the patients with FXS showed the most disrupted sleep microstructure when their sleep architecture and NREM sleep alterations were analyzed by means of the Cyclic Alternating Pattern (42).

### Fragile X associated tremor/ataxia syndrome

This is a different rare condition related to FXS (OMIM #300623) affecting elderly male carries with <200 CGG repeats (premutation). The patients suffer from late onset progressive ataxia, intentional tremor, and cognitive decline. Female carriers are very rarely affected (43). In a relatively recent study from a center specializing in FXTAS, history compatible with OSA was looked for during the clinical evaluation and when positive the results of NPSG were obtained from the physicians who had referred the patient for a sleep study. Among 118 patients with FXTAS, 31.4% suffered from OSA as compared to 13.8% of 174 patients with the premutation but without FXTAS and 8.6% of 174 normal controls (44).

### Angelman syndrome

This AR neurodevelopmental disorder, known also as the “happy puppet syndrome,” consists of craniofacial dysmorphism, microcephaly, severe mental and speech retardation, ataxia of gait, puppet-like jerky movements, bouts of involuntary laughter, and epilepsy. In the majority of the patients, there is an absence of a maternal contribution to the imprinted region on chromosome 15q11–q13 (OMIM #105830). Sleep/wake rhythm disorders, multiple nocturnal awakenings, and difficult sleep onset are reported in 20–80% of the patients. The characteristic published NPSG findings in Angelman syndrome (AS) are based on a small number of patients. When compared to children with primary mental retardation with and without epilepsy, 14 children with AS showed higher percentage of wakefulness after sleep onset, awakenings from sleep, and a longer sleep and REM latencies. PLM was quite frequent in both AS and the non AS retarded subjects. SBD was present in AS and its severity as judged by AHI >5 was not significantly different in the three examined groups. It was concluded that the sleep abnormalities recorded in AS are mostly related to mental retardation, epilepsy, and its drug treatment (45).

### Prader–Willi syndrome

Prader–Willi syndrome (PWS) (OMIM #176270) is a rare AD disorder occurring in 1:10,000–1:25,000 live births. It is caused by deletion or disruption of a gene or several genes on the proximal long arm of the paternal chromosome 15 or maternal uniparental disomy 15. The clinical characteristics consist of diminished fetal activity, infantile hypotonia and failure to thrive. Somewhat later progressive significant weight gain due to ravenous appetite resulting in morbid obesity, short stature, small hands and feet, hypogonadotropic hypogonadism and mental retardation are recognized (46). SBD in the form of OSA is quite common. Sleep is often impaired leading to excessive daytime sleepiness in about 70–85% of the patients (47). Based on MSLT results, 40–50% of adult patients suffer from severe daytime sleepiness (48). Abnormal sleep consists of shortened latency and duration of REM sleep, sleep-onset REM periods, OSA, and sometimes “narcoleptic like” symptoms. Recently, Sedky et al. (49) reviewed 14 studies on 224 children with PWS who underwent NPSG as part of an assessment of OSA. In 179 (79.91%), the diagnosis of OSA was confirmed. Severe OSA was present in 24.58% of the adolescent patients. Narcolepsy was diagnosed in 35.71% of the study population. Adenotonillectomy was helpful but residual OSA was still present post-operatively. The etiology of impaired sleep in PWS is probably multifactorial considering the close association of obesity and OSA. However, an additional role of hypothalamic dysfunction present in PWS cannot be ruled out.

### Williams–Beuren syndrome

This AR neurodevelopmental disorder (OMIM #612547) is the result of a microdeletion at a specific region of chromosome 7. The prevalence of Williams–Beuren syndrome (WBS) was estimated at 1:7500. The affected children suffer from neurological, cognitive, cardiovascular, musculoskeletal, and endocrine abnormalities. Mild-moderated mental retardation and symptoms similar to those of children with attention deficit hyperactivity disorder (ADHD) are the main neurocognitive characteristics. Parents are frequently bothered by their child’s disordered sleep. Mason et al. (50) studied 35 youngsters aged 2–18 years with sleep and behavioral questionnaires and NPSG. The findings were compared to those obtained from matched healthy subjects. The main symptoms reported were difficulty falling asleep, increased restlessness, and increased respiratory-related arousals. NPSG indicated decreased sleep efficiency, increased time spent awake after sleep onset and increased slow wave sleep. There was a good correlation between parental ratings of arousals/awakenings during the night and the NPSG findings. The authors were unable to confirm earlier observations of increased rate of PLM in their patients. Although 52% of their subjects showed symptoms compatible with ADHD, which could explain most of the findings, there was no clear cut association between parental report of “ADHD like” symptoms and the sleep disturbances revealed in this particular large and recent study.

### Smith–Lemli–Opitz syndrome

This AR rare syndrome (OMIM #270400) affects 1:20,000–80,000 live born American Caucasian infants who present with variable clinical features and severity (51). The typical phenotype is a child with characteristic peculiar facial appearance, cleft palate, microcephaly, hypotonia, cardiac defects, prenatal and postnatal growth retardation, postaxial polydactyly, two to three toe syndactyly, and hypogenitalism. Malformations of the brain and lung are less common (52). Rarely, patients may present with minor anomalies and enjoy almost normal development (53). Smith–Lemli–Opitz syndrome (SLOS) is caused by deficiency of 7-dehydrocholesterol (7-DHC) reductase, encoded by the DHCR7 gene located on chromosome 11. As a result, cholesterol synthesis is impaired leading to marked hypocholesterolemia (54). In 1998, an abnormal sleep pattern was reported in a significant number of affected children diagnosed on clinical basis (53). More recently, a systematic evaluation of sleep characteristics in 18 patients with biochemical confirmation of the diagnosis disclosed the presence of nocturnal snoring in half of the patients, labored breathing in 11%, a variety of abnormal sleep behavior abnormalities, and excessive daytime sleepiness (51).

It is intriguing to postulate that the low serum cholesterol in patient with SLOS may play a role in their impaired sleep. This assumption can be ruled out as none of our 14 young patients with marked hypocholesterolemia due to abetalipoproteinemia (Bassen–Kornzweig Syndrome – BKS, OMIM #200100) complained of impaired sleep. Moreover, a literature search using the key words, hypercholesterolemia, BKS and sleep, failed to reveal such an association. The presence of facial anomalies, cleft palate and muscle hypotonia could contribute to snoring and breathing abnormalities mentioned above. It should be noted that without objective studies such as NPSG, the complete spectrum of sleep impairment in SLOS cannot be fully determined.

### Segawa’s disease

This is treatable form of AD hereditary dystonia associated with a unique sleep disorder (OMIM #605407). The disease is caused by a large number of mutations at the guanosine triphosphate cyclohydrolase 1 (GTPCH1) gene located on chromosome 14 (55). The main clinical characteristics are progressive postural dystonia with marked diurnal fluctuation. While during the early morning hours there is only a hint of dystonia, it is getting progressively pronounced and disabling, reaching a peak of severity during the early afternoon. The ongoing dystonia is accompanied by uncontrolled urge to sleep. In fact, one of our affected girls used to fall asleep at about 5p.m. and woke up at about 3–4a.m. to the dismay of the other family members. The motor disability is not associated with mental or primary psychological abnormalities or features of Parkinsonism. Administration of small doses of l-DOPA results in almost complete normalization of the motor impairment as long as treatment is given. One of our patients successfully treated for 37 years with small doses of Dopicar^®^ and later Sinemt^®^ had not shown any of the well-known disabling side effects encountered especially in patients with early onset Parkinson disease (56). Interestingly, the sustained hypersomnolence that she experienced during childhood before the initiation of drug treatment which improved her sleep and vigilance, was replaced during adolescence and adulthood by severe insomnia and numerous awakenings during the night. The inability to maintain sufficient nocturnal sleep was resistant to treatment with a large number of hypnotics, alterations in the dose of l-DOPA, administration of different dopaminergics and a variety of SSRI type antidepressants. A repeated NPSG was consistent with reduced sleep efficiency due to frequent awakenings from sleep stage 2. The results of PSG evaluations in patients with Segawa’s disease (SD) are variable. This may be due to the natural course of the disease during which the diurnal variations in sleep ameliorate with age or due to genetic heterogeneity caused by the numerous mutations at the gene for SD. Segawa (57) showed with selective sleep stage deprivation studies that twitch movements (TM) during REM sleep were reduced by 20%, perhaps related to the decreased dopaminergic activity in those patients. An improvement in the impaired sleep symptoms concomitantly with an increase in TM was noted with l-DOPA. All other parameters of the NPSG were normal. Gadoth et al. (58) have assessed the number of body movements (BMs) during NPSG recording in 3 girls with the sporadic form of SD (two off and one on l-DOPA) and 11 of their healthy first degree relatives. Increased number of BMs was found in the three girls. Both parents of one girl had increased BMs while six of the parents and three of the siblings had PLM. In 18 patients with genetically confirmed SD reported by Van Hove et al. (59), subjective daytime sleepiness, urge to oversleep and frequent nightmares were noted. However, both NPSG and MSLT were normal except for shorter total REM sleep, which was attributed to medication effect.

### Sepiapterin reductase deficiency

This AR form of DOPA responsive movement disorder (OMIM #182125) is caused by mutations in the sepiapterin reductase (SPR) gene, located on chromosome 2. The encoded SPR participates in the tetrahydrobiopterin recycling pathway involved in the biosynthesis of dopamine and serotonin (60). Symptoms appear very early in life and may be erroneously diagnosed as cerebral palsy. The correct diagnosis becomes evident somewhat later when motor and speech delay, axial hypotonia, dystonia, weakness, and oculogyric crises with diurnal fluctuation and sleep benefit are detected. In addition, psychomotor impairment slowly develops. A striking feature is severe daytime sleepiness (61). In a 28-year-old patient with sleep abnormalities since early childhood, reduced metabolism of serotonin in the cerebrospinal fluid and flat serum melatonin profile were found. The patient underwent a sleep interview, wrist actigraphy, sleep log over 14 days and 48 h continuous sleep, and core temperature monitoring. Mild hypersomnia with prolonged sleep time (704 min) and ultradian sleep–wake rhythm with sleep occurrence every 11.8 ± 5.3 h were documented. Supplementation with 5-hydroxytryptophan resulted in improved cognition as a consequence of reduction of total sleep time to 540 min and restoration of serotonin metabolism in the CSF, serum melatonin profile, and circadian sleep–wake rhythm (62).

## Autonomic Dysfunction

### Familial dysautonomia

Familial dysautonomia (FD), known also as Hereditary Autonomic and Sensory Neuropathy type III (HASN III) (OMIM #223900), is a rare AR disorder affecting exclusively infants and children of Jewish Ashkenazi origin with a carrier rate of 1:30, which increases to 1:18 in families of Polish origin (63). It is caused by a specific splicing mutation in the IKBKAP gene located on chromosome 9. The discovery of this mutation made it possible to reach an accurate prenatal diagnosis, which resulted in a dramatic reduction in the number of new patients. The pathophysiology of FD is attributed mainly to a progressive autonomic neuropathy associated with progressive loss of small myelinated, and to a lesser extent, unmyelinated fibers. The clinical manifestations may be evident at birth and with time the affected children and young adults suffer from cardiovascular, respiratory, gastrointestinal, musculoskeletal, and renal dysfunction. In addition, marked emotional and behavioral adaptive problems are present (64). Breathing abnormalities in the form of breath holding spells appear during infancy and persist throughout life. There is an outstanding ability of the affected children to hold their breath for extended periods of time, which may be responsible for the high rate of premature sudden death. Both phenomena are the result of defective responsiveness of the brainstem chemoreceptors to changes in PaO_2_ and PaCO_2_. Severe progressive kyphoscoliosis and cardiac dysatonomia are significant contributing factors to the presence of SBD. In addition and probably related are difficulties in sleep setting and awakening in the morning. Among 13 patients, 5–31 years of age, breath holding spells and difficulties in waking in the morning were reported by 69% and excessive daytime sleepiness by 38%. NPSG disclosed the presence of obstructive and central apneas mainly during stage 2 and REM sleep and also during sleep stage 3–4 (65).

### Congenital central hypoventilation syndrome

This is a rare life-threatening AD disorder of central autonomic control of respiration (OMIM #209880). Clinical presentation occurs usually at birth or soon after. Known previously as “Ondine’s curse,” it is characterized by severe reduction in ventilatory sensitivity to hypercapnia both during NREM slow wave sleep, REM sleep, and wakefulness. The diagnosis requires the presence of marked hypoventilation during sleep without primary neuromuscular, cardiac, respiratory, or brainstem disease. Autonomic dysfunction outside the central nervous system is also present in the form of Hirschsprung disease, cardiac arrhythmia, pupillary abnormalities, excessive sweating, and tumors of neural crest derivatives (66). In more than 90% of the cases, a poly-alanine repeat expansion due to mutations in the paired-like homeobox PHOX2B gene located on chromosome 4 is present (67). Although most of the cases present during early infancy and sometimes at birth, clinical signs may appear at an older age. The clinical presentation as well as the phenotype–genotype correlations are variable. Thus, the disease should be considered in young individuals with life-threatening paroxysms of cyanosis during sleep, unexplained seizures, respiratory depression induced by anesthesia, sedation or anti-convulsive drugs, cognitive impairment with previous cyanotic spells, primary nocturnal hypoxemia associated with hypercarbia, superior ability to stay underwater, recurrent pneumonia, and sudden unexplained childhood/infant death. The exact relation between sleep stage and the timing of hypercarbic/anoxic events is of interest, but such data are unfortunately not available. We were able to find a single comment on PSG done in one patient, which was summarized as “significant oxygen and carbon dioxide abnormalities occurring after sleep onset” (68).

### Sleep in hereditary skeletal deformities

Dwarfism and gigantism are frequently associated with craniofacial dysmorphism and neurological impairment, both major risk factors for sleep abnormalities. Out of numerous syndromes comprising this group, Achondroplasia and Marfan syndrome (MS) are the best studied in regard to sleep and breathing characteristics during sleep.

### Achondroplasia

This AD syndrome (OMIM #100800) is the most common form of short limb dwarfism with a prevalence of 1:1000–30,000 in newborns (69). It is caused most commonly by a mutation in the fibroblast growth factor receptor-3 (FGFR-3) located on chromosome 4. Sleep characteristics gained special interest since those patients are prone to UA obstruction during sleep as well as to impairment of central respiratory control due to impingement of the lower brain stem and medulla within the narrow foramen magnum. OSA is present in 10% of affected children aged 4 years, and in 16% of all patients regardless of age (70). Between 10 and 85% of affected infants and children seek treatment for major respiratory difficulty, i.e., OSA, waking cyanotic episodes, and chronic respiratory insufficiency. The predisposing factors are thoracic cage restriction and midfacial hypoplasia. In addition, foramen magnum stenosis caused by dysplastic basiocciput, occipital bone, and craniovertebral junction with secondary brain stem compression, may contribute to dysregulation of breathing. Recently, the sleep characteristics of 29 children and adolescents (median age: 3 years, range 0.4–17.1) were reported. A third underwent UA surgery previous to the study and 17% had a previous craniocervical decompression for foramen magnum stenosis. Habitual snoring was reported in 77% and witnessed sleep apnea in 33%. Nocturnal PSG was considered abnormal in 93% and AHI was ≥10 in 43%. The majority of apneas were obstructive with 22% central and 5% mixed type. The correlation between symptoms of SBD and the results of NPSG was poor; however, AHI correlated well with severity of daytime fatigue (71).

### Marfan syndrome (MS)

Marfan syndrome is a rare AD disorder of connective tissue (OMIM #154700) due to mutations in the gene encoding for fibrillin-1 (FBN1) located on chromosome 15. The frequency of MS is 0.01% in the general population. Although connective tissue is globally affected, the clinical manifestations involve the eyes, lungs, thoracic aorta, dura matter, and skeleton. The main cause for early mortality is dissection and rupture of the aorta. Sleep complaints such as snoring, apnea, sleep disruption, daytime fatigue, and difficulties in memory and concentration are common (72). Craniofacial anomalies such as retrognathia, increased mean neck circumference, and increased nasal airway resistance were considered as causing OSA in MS. Interestingly, in a recent study on a relatively large group of unselected patients, sleep apnea was found in about 30%, however, about 50% of the apneas were central rather than the expected obstructive apneas (73). Considering the above, it is logical to screen patients with MS for SBD regardless of the presence of the accepted clinical clues for OSA.

### Treacher Collins syndrome

This rare AD disorder (OMIM #248390) is one of the several congenital craniofacial anomalies such as Pierre Robin, Apert, and Crouzon syndrome, which are associated with severe OSA. Patients with Treacher Collins syndrome (TCS) carry mutations in the TCOF1 gene, located on chromosome 5. The classical clinical features are micrognathia, zygomatico-temporo-maxillary dysostosis, mandibular hypoplasia, choanal atresia, underdevelopment of the auricles, down slant of the eyelids, coloboma of the eyelids, and hypoplasia of the zygomatic bone and lateral orbital wall. The incidence of TCS is 1:50,000 live births. In about 60% of the patients, the family history is negative. The abnormalities mentioned above explain the high frequency of OSA in those patients, which ranges from 54% in children to 41% in adults (74). Surgical relief of airway obstruction is complicated due to the numerous locations of possible obstruction. Thus, a careful determination of the most useful site (sites) for reconstructive surgery is the key to successful outcome.

### Sleep in hereditary neuromuscular disorders

In general, patients with hereditary neuromuscular disorders (HND) present during childhood and are at risk for sleep-related hypoventilation leading to sleep hypoxemia, pulmonary hypertension, and secondary cognitive impairment. SBD is not rare manifesting as severe impairment of respiratory regulation during sleep. In addition to OSA, some children with HND suffer from reduced sensitivity of their central chemoreceptors, which contributes to SBD. Considering the fact that the largest load of patients with genetically determined neurometabolic disorders are those with neuromuscular diseases, which are frequently complicated by SBD, it was interesting to estimate its frequency in a busy neuromuscular clinic. Labanowski et al. (75) have performed such a study in the New Mexico Neuromuscular Disorders Clinic. Out of a total of 306 patients, 54 adults and 10 children agreed to participate in the study. Of those, 42% suffered from SBD. The presenting symptoms of SBD in children with HND usually manifest when muscle weakness is still mild. At that stage, there are no clinical signs of respiratory difficulty during the day or they are so mild and therefore are often masked by the general concern about the decreased muscle strength. The children may present with daytime sleepiness and fatigue, reduced sleep quality, frequent awakenings, morning headaches, mood changes, attention and learning deficits, failure to thrive and more rarely, nocturnal hypoxic seizures. If unrecognized, progression to secondary polycythemia, hypertension, and heart failure is inevitable (76).

### Duchenne muscular dystrophy

Duchenne muscular dystrophy (DMD) (OMIM #310200) affects boys and very rarely girls. The affected carry mutations in the dystrophin gene located on chromosome X, which is responsible for progressive degeneration of muscle fibers. Subsequently, the affected subjects will become wheel-chair bound at the age of 10–12 years with a life expectancy of 26 years. Dilating cardiomyopathy occurs in almost all patients and is the cause of early death in 10–20% (77). In addition to limb-girdle musculature, there is a progressive loss of respiratory muscle fibers leading to ineffective cough, decreased ventilation, recurrent pneumonia, atelectasis, and respiratory failure during wakefulness and especially during REM sleep. Respiratory problems are first manifested during sleep and if detected early and given appropriate treatment, the patients will enjoy an improved life quality, which is very significant considering the severity and fatal nature of the disease. SBD is usually present when forced vital capacity (FVC) is reduced to 65%, thus FVC monitoring can be useful for appropriate timing of PSG. The appearance of nocturnal hypoventilation predicts the onset of progressive daytime hypercapnia some 2 years later. The institution of non-invasive ventilation (NIV) before the development of daytime hypercapnia is highly recommended (78). Annual evaluation of SBD should be initiated when the patient becomes wheel-chair bound. Dilated cardiomyopathy, which primarily affects the left ventricle is the cause of death in about 10–20% of the patients. There is a vicious cycle of events finally leading to right ventricular failure. Initially, left dilated cardiomyopathy appears accompanied by dyspnea, which subsequently evolves to central apnea. The progression of respiratory difficulties will lead to right ventricular failure accompanied by pulmonary hypertension and respiratory failure. To delay the appearance of this vicious cycle, the institution of non-invasive ventilatory support should be considered when hypercapnia (pCO2 ≥50 mmHg) and/or hemoglobin saturations remains <92% when awake. Most of the patients will benefit from NIV while tracheostomy is only rarely justified. Interestingly, “preventive” nocturnal non-invasive positive pressure ventilation (NIPPV) should be avoided because patients who received NIPPV had a decreased survival rate as compared to the untreated patients (79). Somewhat similar features may be present in other HND such as the severe form of limb-girdle, facio-scapulo-humoral, Emery–Dreifuss, and congenital muscular dystrophies.

### Myotonic muscular dystrophy type 1 (MMD-1)

This AD multisystem disorder (OMIM #160900) is the most common form of muscular dystrophy in adults. It is caused by a heterozygous trinucleotide (“Triplet”) repeat expansion (CTGn) in the 3′-untranslated region of the dystrophia myotonica protein kinase gene on chromosome 19. The number of the triplet repeats predicts the age of onset and eventually the severity of the disease. Weakness, wasting and myotonia of the facial, oropharyngeal, lingual, masticatory, and distal muscles are the characteristic neuromuscular abnormalities. Daytime hypersomnolence is the most common sleep-related complaint being present in 70–80% of patients. The main PSG findings include obstructive and central apnea during sleep and wakefulness, PLM, and REM sleep dysregulation. Symptoms of restless leg syndrome (RLS) are frequently reported (80). Interestingly, some patients who were adequately treated for SBD are still sleepy during the day. In those patients administration of Modafinil (Provigil^®^) can be valuable for improving daytime vigilance (81). When the disease manifests during childhood and the muscular symptoms are accompanied by daytime somnolence which is frequently associated with learning disability, PSG is indicated to detect the presence of OSA and PLM which are present in about 65% of this particular population (82). The congenital onset of the disease is the severest form of MMD-1 manifesting severe skeletal, neuromuscular and cognitive abnormalities. Thoracic muscle weakness and abnormal craniofacial features are the cause of increased UA resistance associated with mixed central and obstructive apnea (83).

### Myotonic muscular dystrophy type 2

This relatively rare AD subgroup of MMD (OMIM #602668) is less severe than MMD-1. It is characterized by proximal leg and finger flexor weakness, pain, and stiffness, without significant clinical myotonia, which is clearly present on electromyography. Patients with myotonic muscular dystrophy type 2 (MMD-2) have only mild facial weakness, ptosis, and cognitive impairment associated with white matter abnormalities. Respiratory muscles are not significantly affected while the proximal leg and distal hand muscles, in particular the finger flexors, are early involved. The disease is caused by a mutated ZNF9 (zinc finger protein 9) gene located on chromosome 3, resulting in a CCTG unstable repeat expansion. The repeat length is not a predictor of age of onset and severity of symptoms as in MMD-1. Sleep characteristics were only recently studied in eight patients. Excessive daytime sleepiness was reported by six patients, insomnia by five, excessive fatigue by four, and features compatible with RLS by four. NPSG was recorded in five and showed the presence of OSA in three (84).

### Spinal muscular atrophy

This AR disorder affects infants, children, and adults. Four subtypes (I-IV) of spinal muscular atrophy (SMA) are recognized. Types I–II present during infancy, type III during adolescence and type IV during adulthood. The progressive death of spinal motor neurons associated with symmetrical muscle wasting and weakness is caused by deletions and only rarely by mutations in the survival motor neuron 1 (SMN1) gene located on chromosome 5. SMA I, known also as Wording–Hoffman disease (OMIM #253300), is the second most common neuromuscular disorder in children affecting 8:100,000 live births. It is also the second in-line lethal condition of childhood after cystic fibrosis. The clinical onset becomes evident at about 3 months of age and sometimes even earlier. Death mainly from pneumonia occurs at about the age of 2 years. Hypercapnia, somnolence, morning headaches, and attention deficits are seen in children with SMA I–II. They suffer from hypoventilation due to respiratory muscle weakness and characteristic paradoxical breathing expressed as thoraco-abdominal asynchrony, which is mostly evident during quite and active sleep. There are only few reports on nocturnal sleep characteristics and the effect of NIV on those characteristics. Night sleep was studied by NPSG in seven children with SBD, six with SMA I, and one with SMA II. NPSG prior to initiation of NIV showed an increase in sleep stage 1, 2 concomitant with decreased stage 3, 4 and REM sleep. Numerous arousals and decreased sleep efficiency were also noted. Following NIV, the duration of sleep stage 1, 2 decreased concomitant with an increase in sleep stage III–IV, a tendency toward longer REM sleep, and a significant decrease in the number of EEG-arousals (85). The exact timing for institution of NIV is not yet established as routine respiratory function studies are usually difficult and annual PSG or overnight oximetry are not always available. The emerging possibilities of genetic manipulation of SMA using Antisense oligonucleotides, which is useful in the mouse model of SMA (86) may reinforce the need for early detection and treatment of respiratory failure and SBD in those patients.

### Hereditary cerebellar degeneration

The cerebellum and its central and peripheral connections are involved not only in motor regulation but also in certain cognitive functions as well as in sleep. Out of the heterogeneous hereditary cerebellar degeneration (HCD), those with significant sleep impairment are the dominant spino-cerebellar atrophies (SCA), in particular type 1, 2, 3, 6. SCA3, known also as Machado–Joseph disease (OMIM #109150), is the most common AD ataxia. It is caused by a trinucleotide (CAG n) repeat expansion encoding glutamine repeats in the ataxin-3 gene (ATXN3) located on chromosome 14. In addition to ataxia, the patients suffer from motor neuron disease, external ophthalmoplegia, Parkinsonism, dystonia, peripheral neuropathy, and non-motor symptoms such as olfactory, memory, executive, and psychiatric impairment. Complains of insomnia, excessive daytime sleep, RLS, REM sleep behavior disorder (RSBD), and sleep apnea are quite frequent. In a recent clinical and PSG evaluation of 22 patients with average disease duration of 7.2 ± 4.6 years, PLM was recorded in 77, REM sleep without muscle atonia in 73%, RSBD in 59%. 54% reported symptoms compatible with RLS (87). Somewhat similar results were recently reported in 16 Chinese patients (88).

## Congenital Neuropathies

### Hereditary motor and sensory neuropathy

Hereditary motor and sensory neuropathy (HMSN), known also as Charcot–Marie–Tooth disease (CMTD), is a group of inherited neuropathies, which differ in their clinical, electrophysiological, pathological, and genetic features. The vast majority of patients with AD HMSN carry a mutation responsible for a duplication of the gene encoding peripheral myelin protein-22 (PMP 22), located on chromosome 17 (HMSM-1A, OMIM #118220). The prevalence of CMTD is 1:2500 (89). Sleep abnormalities in the form of OSA and PLM are well known in CMTD. In a quite recent sleep study on 61 patients with CMT1 (dominant HMSN), 34 suffered from HMSN-1A, 10 from HMSN-1B (OMIM #118200) caused by heterozygous mutation in the gene encoding myelin protein zero (MPZ) located on chromosome 1, and 17 had HMSN-X1 (OMIM #302800) caused by hemizygous or heterozygous mutation in the GJB1 (Gap Junction Beta 1) gene located on chromosome X. All patients had subjective reduced nocturnal sleep quality, daytime hypersomnolence, and significant fatigue. NPSG disclosed the presence of OSA in 37.7% of all patients. Interestingly, the severity of OSA was better correlated with the functional disability of the patients rather than with BMI and aging. A significant portion of patients had mixed apnea. It was suggested that the particular tendency for OSA in those patients may be related to spread of the neuropathy to the pharyngeal muscles (90).

### Congenital myopathies

This heterogeneous group of non-dystrophic early onset myopathies is characterized pathologically by presence of protein aggregates within muscle fibers. Molecular studies proved that those disorders are expressed as mild to severe phenotypes with adolescent and even late adult onset. In this heterogeneous group of disorders, infants and children with nemaline, myotubular, centronuclear, and protein aggregate myopathies suffer from SBD due to deformities of the facial bones, severe scoliosis, and marked weakness of the facial, bulbar, and respiratory muscles.

### Nemaline myopthy

Nemaline myopthy (NM) in its severe form presents at birth with marked hypotonia, dysphagia, and dyspnea. However, even the “classical” form may present at birth, during childhood, and even early adulthood with milder clinical features. The determinant of severity is the degree of scoliosis and restrictive lung disease. The clinical variability of NM is probably due to the fact that the disease is caused by seven different gene mutations known today. Of those, the most common is a mutation in the nebulin gene located on chromosome 2. The clinical heterogeneity was demonstrated in four patients reported by Sasaki et al. (91). One of the studied patients suffered from the severe infantile form and the other three from the benign “classical” form. While the child with the severe form had breathing abnormalities at birth, the other three developed an acute respiratory crisis at the ages of 2, 8, and 9 years when they were still ambulant and muscle weakness was mild. This discrepancy seems to be rather the rule in NM. Thus, careful monitoring of respiratory functions is required especially when such patients show a discrepancy between motor and respiratory functions. In the above mentioned publication, NPSG was performed in the child with the severe form and in one of the three with the mild classical form. Apneic spells or irregular thoracic movements associated with hypercapnia were recorded only during REM periods in both children. As mentioned above, patient with myotubular, centronuclear, and protein aggregate myopathies are not very different in terms of SBD, which is related to the severity and distribution of muscle weakness.

## Closing Remarks

Although only a portion of all hereditary disorders, which are complicated by sleep and often breathing problems have been included in this review, we believe that the entities described represent well the scope of those problems and will provide the reader who may be asked to evaluate such patients with clues to “ when and how” should sleep problems be approached. Moreover, detecting and treating sleep problems in those patients may improve their already impaired life quality.

## Conflict of Interest Statement

The authors declare that the research was conducted in the absence of any commercial or financial relationships that could be construed as a potential conflict of interest.
