# Corrigendum: Sleep and Sleep Disorders in Rare Hereditary Diseases: A Reminder for the Pediatrician, Pediatric and Adult Neurologist, General Practitioner, and Sleep Specialist

**DOI:** 10.3389/fneur.2015.00006

**Published:** 2015-01-26

**Authors:** Natan Gadoth, Arie Oksenberg

**Affiliations:** ^1^Sleep Disorders Unit, Loewenstein Rehabilitation Center, Raanana, Israel; ^2^Department of Neurology, Mayanei Hayeshua Medical Center, Bnei Barak, Israel; ^3^Sackler Faculty of Medicine, Tel-Aviv University, Tel-Aviv, Israel

**Keywords:** Angelman, Prader-Willi, Williams-Beuren, syndrome, Chromosome Mapping

A corrigendum on Angelman, Prader – Willi and Williams-Beuren Syndromes (Page 5)

Angelman and Williams Beuren syndromes are not autosomal recessive (AR) and Prader-Willi syndrome is not autosomal dominant as incorrectly stated in the text.The rest of the molecular genetic information given is correct. We apologies for the inconvenience.

## Conflict of Interest Statement

The authors declare that the research was conducted in the absence of any commercial or financial relationships that could be construed as a potential conflict of interest.

